# eGenomics: Genomes and the Environment

**DOI:** 10.1002/cfg.493

**Published:** 2005

**Authors:** Dawn Field, Bela Tiwari, Jason Snape

**Affiliations:** 1 Molecular Evolution and Bioinformatics Section and NERC Environmental Bioinformatics Centre Mansfield Road Oxford Centre for Ecology and Hydrology Oxford OX1 3SR UK; 2 Brixham Environmental Laboratory AstraZeneca Devon TQ5 8BA UK


**Comparative and Functional Genomics**
*Comp Funct Genom* 2005; **6**: 357–362.
Published online in Wiley InterScience (www.interscience.wiley.com). **DOI:** 10.1002/cfg.493

**Meeting Report**

### eGenomics: Genomes and the Environment

Dawn Field*[1], Bela Tiwari[1] and Jason Snape[2]
[1] *Molecular Evolution and Bioinformatics Section and NERC Environmental Bioinformatics Centre, Mansfield Road, Oxford Centre for Ecology and Hydrology, Oxford OX1 3SR, UK*
[2] *Brixham Environmental Laboratory, AstraZeneca, Devon TQ5 8BA, UK*

**Correspondence to: Dawn Field, Molecular Evolution and Bioinformatics Section and NERC Environmental Bioinformatics Centre, Mansfield Road, Oxford Centre for Ecology and Hydrology, Oxford OX1 3SR, UK. E-mail: dfield@ceh.ac.uk*

Received: 30 September 2005
Revised: 3 October 2005
Accepted: 4 October 2005

**Keywords: genomes; environment; bioinformatics; data management; metagenomics; microarrays; metabolomics; data standards**

As concerns over climate change, pollution and other anthropogenic changes to the environment increase, interest in finding new solutions for understanding and mitigating these conditions is growing. The emerging discipline of environmental genomics investigates how living organisms adapt to and are impacted by their environments, using genomic technologies. The UK Natural Environment Research Council (NERC) funds environmental genomic research and has recently invested over £26 million in two science programmes in this area: 'Environmental Genomics' and 'Post-Genomics and Proteomics'. To support researchers working in this area, NERC has created the NERC Environmental Bioinformatics Centre (**NEBC**)[1] (Field *et al.*, 2005) and continues to fund a range of data centres, including the British Oceanographic Data Centre (**BODC**)[2], and allied initiatives that are starting to deal with molecular data in addition to traditional environmental data. To provide improved access to 'omic' technologies, NERC is also investing in **molecular genetic facilities** [3]. All of these activities are complemented by an investment in environmental eScience, which includes funding of the NERC Data Grid (**NDG**) [4] and the establishment of the National Institute for Environmental *e*Science (**NIE*e*S**) [5].

Given the increasing investment in this area, we organized a workshop entitled 'eGenomics: Genomes and the Environment' to strengthen the potential interactions between these members of the NERC community. The workshop took place at NIE*e*S in Cambridge, UK, on 5–6 September 2005. This event brought together researchers, bioinformaticians, computing specialists and data managers to present their current work and discuss the challenges and opportunities associated with merging genomic and environmental data. This workshop was organized into sessions dedicated to research, data standards, data management and discussion.

### Session I: Finding and characterizing genes and genomes in the environment: metagenomics

This session focused on the use of metagenomics in characterizing communities of increasing complexity (extreme environments, oceans and soils).

Copyright © 2006 John Wiley & Sons, Ltd.

**Francisco Rodríguez-Valera** (Universidad Miguel Hernández) delivered the plenary talk of the workshop, describing his group's work on an extreme halophile community living 'on the edge of life' in saturated salt brines (in salterns). He opened the talk with a slide showing his young son standing before a huge telescope and made the analogy that we are also like children standing before a powerful new tool that we do not yet know how to use. We have a powerful technology in the form of metagenomics; we just have to learn where to point it and how to use it. His work demonstrates that the study of simple, well-characterized communities is an excellent place to start. He discussed how the two dominant species from his community, *Haloquadratum walsby* (strain HBSQ001 is 80% of the community) and *Salinibacter ruber* (which is 15% of the community), have recently been isolated (Bolhuis *et al*., 2004) and sequenced. A metagenomic study of this saltern habitat has further shown that, while *H. walsby* appears clonal according to 16S ribosomal RNA studies, the sequenced strain represents only a fraction of the total gene pool for this species. He also used the genomes of these two species to illustrate the phenomenon of the habitat gene reservoir, reporting that not only phylogeny, but also *habitat* determines what genes a bacterium will hold.

The next speaker, **Ian Joint** (Plymouth Marine Laboratory) reminded the group that few environments are as simple as salterns by introducing a project aimed at using metagenomics and microarrays to characterize microbial communities in aquatic environments. Specifically, this large collaborative project hopes to test the hypothesis that microbes exist in definable communities in aquatic environments, and that this structure impacts their functional roles. Ian used the apt phrase 'nothing stands still' to describe aquatic habitats, and stressed the importance of collecting and maintaining accurate environmental descriptions of samples. He also made the point that we need to build archives of physical samples, a more difficult and expensive undertaking than that of archiving data. As well as supporting Francisco's call for hypothesis-driven science, Ian stressed the need to create inclusive databases to facilitate the comparison of data.

**Michael Barton** (University of Newcastle) described a project driven by the need to reconstruct metabolic pathways in metagenomic datasets that is set to change the face of computing at the University of Newcastle. He and his supervisor **Anil Wipat** are using the 'Metabolic Search and Reconstruction Kit' (Metashark) [6] (Pinney *et al*., 2005) to identify enzymes in metagenomic datasets in the hope of understanding the functions of microbial communities. 'Parallel shark hunts' (or computationally intensive searches of raw sequences using enzyme profiles) are run over the condor pool, which scavenges spare CPU cycles from available workstations. The pool is set to eventually unify all 10 000 Linux and PC workstations on campus into a single cluster.

The session chair, **Andy Johnston** (University of East Anglia), wrapped up this lively session with a talk on the issue of recognizing and dealing with sampling bias in metagenomics. His group has recently built a metagenomic library from a waste water treatment plant by inserting DNA into a broad-host range cosmid, using *Rhizobium* as a host in the hopes of characterizing the *nif* nitrogen fixing genes. This cloning approach increases the likelihood of the expression of foreign genes, as this host has 20 sigma factors and so can express more foreign genes than *E. coli*. Surprisingly, though, he found that the library appeared to lack any copies of the 19 available *nif* genes that could correct the corresponding $Nif^-$ mutants of a range of different bacteria. He further found that nitrogen fixation genes are only found in 1 of the 4 largest marine metagenomic datasets published to date (the fourth one he examined) (Johnston *et al*., 2005). His take-home message from this experience was that metagenomic studies represent only a single data point, and therefore we risk severe misinterpretation of the data due to sampling bias if we aren't mindful of these limitations. His study illustrates that sampling bias will make it difficult to track down the species/genes responsible for some essential earth system processes and stressed that 'Petri dish approaches' still have an important role to play in delivering high quality results in environmental genomics. For example, there are essential earth system processes, such as the release of dimethyl sulphide (DMS) into the atmosphere from the oceans, that could be studied using traditional, reductionist approaches. It is known that this process is carried out by bacteria that can be easily grown in the laboratory, and yet still we know nothing about the genes involved.

Copyright © 2006 John Wiley & Sons, Ltd. 

In the final talk before lunch, our second international speaker, **Terry McIntyre**, Chief of the Environmental Genomics Program at Environment Canada [7], described the environmental genomic efforts in Canada. Canada is experiencing a shift away from curiosity-based research towards more applied work, in large part due to increasing recognition that microorganisms play a variety of key roles in maintaining healthy ecosystems. He stressed that a limited amount of total research funds go to this increasingly important subject and emphasized his interest in seeing the Canadian, US, and UK environmental genomics communities work together towards shared goals.

### Session II: Molecular basis of phenotypes of environmental and ecological relevance

This session contained a series of contributed talks that highlighted the wide range of taxa, systems and approaches being applied in this community. **Mark Viant** (University of Birmingham) discussed the use of metabolomics, the study of the composite metabolites of a cell or sample, in ecotoxicology. This young field has several advantages in the characterization of environmental samples, including the close relationship between the targets of metabolomic studies (lipids, sugars, etc.) and phenotype. In addition, there is no reliance on the availability of a complete genome sequence — a metabolite in one species (e.g. lactate) is the same across all species. This opens the door for comparative metabolomic studies, especially as this method is high throughput and inexpensive on a per sample basis. Mark is pioneering the use of nuclear magnetic resonance (NMR) to detect metabolic phenotypes of fish at different developmental stages and in response to different toxicants. He stressed that one biomarker is not enough to characterize exposure to an environmental stress. He also emphasized the need to solve issues surrounding the capture and management of metabolomic data and methods for metabolite annotation (identification and description of peaks using uniform concepts). Perhaps the greatest need is for chemical libraries to assist peak identification, as only 5–10% of the proteome can currently be identified by NMR.

**Tamas Dalmay** (University of East Anglia) discussed the early stages of a project designed to test the hypothesis that micro-RNAs (miRNA) play a role in environmental adaptation. miRNAs regulate gene expression by blocking translation at the mRNA level. He reminded the group that this has implications for the interpretation of transcriptomic studies because it means the mRNA might be detectable in a sample, but contrary to expectation the protein may not be expressed. The objectives of this project are to find miRNAs in chicken, *Drosophila* and fish and to obtain profiles from different environments using miRNA arrays. miRNAs that are up- or downregulated between environments (e.g. two different temperatures), and the genes they regulate, will then be characterized.

**Martin Ostrowski** (University of Warwick) discussed the potential for doing large-scale comparative genomics on environmentally important organisms. There will soon be more than 20 genomes from his taxonomic group of interest, the unicellular marine cyanobacterium, *Synechococcus*, including three that have been generated for his group by the Moore Foundation. Of special interest to Martin is the ability to relate genomic information back to complex information on niche partitioning of different clades (such as different geographic locations and depth levels) using molecular probes. Martin stressed the value of high-quality, manual annotation methods, compared to high-throughput, fully automated annotations.

The session ended with a talk from **Anna Goostrey** (Plymouth Marine Laboratory) on transcriptional profiling and SNP analysis of the Pacific oyster, *Crassostrea gigas*. She is studying resistance and susceptibility to summer mortality within the EU project 'AQUAFIRST' [8], a systematic, applied project to find resistance to stress markers and genes expressed in association with disease in selected fish and shellfish. This work is an excellent example of how recently developed 'omic' technologies can be used to understand long-term systems, such as oyster lineages subjected to selective breeding over several decades.

### Session III: Data standards

There is a growing need for data standards in the realm of environmental genomics. Standards minimize duplication, foster collaborative science, realise the potential for comparative genomics,

Copyright © 2006 John Wiley & Sons, Ltd. 

and increasingly compliance is required for publication as journals aim to maintain the quality of their published articles. All four speakers in this session stressed that standards projects should be community-driven. **Joe Wood** (**NEBC**) discussed current efforts to extend the international standard for capturing transcriptomic data. **MIAME/Env** [9] has been created to capture information of most relevance to environmental transcriptomic experiments. Capture of Env information has been facilitated by the distribution of maxdLoad2 (Hancock *et al.*, 2005) on Bio-Linux (Tiwari and Field, 2005). **Dawn Field** (CEH Oxford) described an effort to create a new standard to describe complete genome sequences (Field and Hughes, 2005). This new standard would extend the set of information captured in genome annotations, in particular to capture relevant information about the environmental and ecological context of the genomes that have been selected for sequencing (Field D, Garrity G, Morrison N *et al.*, 2005).

**Jason Snape** (AstraZeneca) discussed the importance of data standards in the context of industry and regulatory policy, especially with respect to the use of microarray technologies in the realm of chemical safety assessment for human and environmental health. Standards build confidence and also ensure that exploratory science can be re-usable. The primary obstacles to the establishment of data standards appear to be researcher perceptions. For example, the 'minimum' required is perceived as too detailed; researchers are frustrated by standards 'creep' (frequent versions), and often argue that dedicating resources to these activities drains funds away from experimental work and represents money that could be better spent on 'science'. Jason suggested that these negative perceptions could be overcome if standards are well integrated with the interests of the community and if it is clearly demonstrated that they could directly benefit from high-quality legacy genomic datasets rich in the appropriate metadata.

**Bryan Lawrence** (British Atmospheric Data Centre) closed this session with a discussion of standardization efforts within the **NDG** [4], an ambitious project to build the foundation for integrating environmental metadata in an eScience context. While this is not a genomics project, it provided an excellent introduction to one possible way to solve the issue of interdisciplinary integration issues. The NDG allows access to datasets that range from terabytes of data from remote sensing, to bytes of hard-won data meticulously collected by researchers in the field. Central to the NDG is a metadata taxonomy, which defines different levels of 'metadata'. Bryan's take-home message to this group was to move towards existing communities and solutions if they exist, and he stressed that this does exist for geospatial datasets. He proposed that the best way to get people to comply with any new standard is to show real-world data. He believes that interoperability can be achieved by identifying what communities have in common and exploiting that commonality. Finally, he feels that a significant part of the solution to getting people to adopt standards is to give due recognition to those who generate the metadata (as opposed to the original data).

### Session IV: Data management

As part of protecting its legacy datasets and assuring the interoperability of future datasets, NERC is investing in the development of thesauri and dictionaries that aid in the integration of datasets from different sources. One project working towards this goal is the Ecological Data Grid [10] project (**EcoGRID**). **Neil Bennett** (CCLRC Daresbury Laboratory) described how this group hopes to integrate the data holdings of the Centre for Ecology and Hydrology using the NDG framework. The first goal of this project is to integrate datasets from the Lakes Database, Environmental Change Network and the Countryside Survey vegetation database.

**Tim Booth** (NEBC) described the development of 'EnvBase: A knowledgebase of environmental genomics'. **EnvBase** [11] contains high-level metadata describing research projects and a description of all data holdings. Links to accession numbers in public repositories provide access to raw data. NEBC staff actively work to curate data and help researchers submit raw data to the appropriate public databases. This is the first catalogue of genomic data to be built by the NERC; Tim is now working to make its contents deliverable through the NDG and is interested in seeing it integrated with a variety of other environmental datasets in the future.

**Gwen Moncoiffe** (**BODC** [2]) spoke about efforts to manage data from NERC's Marine and

Copyright © 2006 John Wiley & Sons, Ltd.

Freshwater Microbial Biodiversity science programme (M&FMB). Molecular data was originally deemed to be outside the remit of this data centre, but it quickly became apparent that there was a high risk of data from genetic samples deposited in Genbank becoming permanently detached from information about their environmental context. Gwen has completed a pilot project in which environmental data from the Ambition Cruise and DNA/RNA data generated from these samples are integrated in a searchable format. From this experience, she maintains that the submission of environmental molecular data to public repositories cannot be considered a sufficient condition for proper data stewardship, but that information must also be submitted to an environmental data curation centre.

### Session V: eScience and GRID solutions

**Stuart Ballard** (NIE*e*S) gave the group an overview of GRID technologies and eScience. **NIE*e*S** [5] is working, along with eight regional and one national eScience centres in the UK, towards the eScience vision of making computing power as freely available as power on an electrical grid. Stuart overviewed the four types of eScience technologies: computing power, data sharing, applications provision and communication. Services provided by NIE*e*S to the NERC community include the ability to fund proposals from the NERC community for training events, workshops and working groups. NIE*e*S also runs a summer school and road shows, and invites visitors to come to the centre to learn about eScience. NIE*e*S serves as a first point of contact for any UK environmental eScience enquiries. Further, Stuart stressed that the current priority of the centre is to directly help environmental scientists incorporate Grid technologies into their research.

To conclude the workshop, **Nicolas Bertrand** (Oxford Centre for Ecology and Hydrology) gave participants the chance to tour the NIE*e*S AccessGrid facility and see a live demonstration of this technology. AccessGrid nodes are a core part of the UK eScience toolkit and provide users with high-powered videoconferencing for *ad hoc* meetings, conferences, seminars or virtual workshops. Despite being mainly used in high-end conferencing suites in academic institutions and companies, Nicolas explained that Access GRID technology can also be scaled down and used on more modest systems (e.g. a laptop equipped with a web camera and an echo-cancelling headset), greatly lowering the cost of holding efficient distributed meetings over the internet.

### Discussion

Throughout the workshop there was a strong sense that we are at the early stages of realizing the full potential of environmental genomics. The group engaged in a protracted discussion of the relative cost : benefit ratio of metagenomic studies and the scientific questions that could be addressed with this approach, weighing the obvious benefits of sampling biodiversity against the prohibitive cost of using metagenomics to test any given hypothesis with statistical rigor (since so many data points would be needed). A requirement echoed by most of the researchers at the workshop is the need for higher quality data, especially in the form of improved genomic annotations. Likewise, there was much interest in seeing the use of standardized approaches in environmental genomic experiments. The group agreed that an excellent investment of effort would be to focus on making all environmental transcriptomic studies as reproducible as, say, DNA typing. The group felt that this could be done but would be very difficult. Such improvements would have a massive impact on the quality of data, thus leading to better research outputs and an ability to use these results in influencing regulatory policy.

Many people reiterated in their talks and the subsequent discussion that this is a data-rich and knowledge-poor discipline. Data integration and accessibility was discussed at length, and it was repeatedly stated that we currently lack a framework in which to store and make sense of this type of data. Participants were concerned about the fractured nature of data, and expressed wishes for a comprehensive portal to all relevant data. This prompted an extended discussion of the difficulty of collecting high-quality, complete datasets, and about ways to convince people to submit data and to comply with standards. Biologists agreed that, in principle, for any effort to work, the flow of information must be managed so that submitters only have to submit once to a single, credible and permanent source. It was mentioned that researchers

Copyright © 2006 John Wiley & Sons, Ltd. 

submit molecular genetic and array data to public repositories in large part only because accession numbers are required for publication, and perhaps similar requirements could be put into place by funding bodies to ensure the submission of other types of data to public repositories.

In summary, there was strong sense that this community is young and vibrant and that great benefits are to be had through increasing interactions between groups working in experimental biology, bioinformatics and data management. The workshop closed with a sense that there are numerous challenges to overcome but that the opportunities make it worthwhile. Presentations and further details of the workshop discussions are hosted at the NEBC website (**http://envgen.nox.ac.uk/workshops/**).

### Acknowledgements

This workshop was funded by the National Institute for Environmental eScience, based on a proposal submitted by D.F. and B.T. B.T. is supported by the NERC Environmental Bioinformatics Centre (FG/G13/18/04).

### URLs

[1] NERC Environmental Bioinformatics Centre: **http://envgen.nox.ac.uk/**
[2] British Oceanographic Data Centre: **http://www.bodc.ac.uk/**
[3] NERC Molecular Genetics Facilities: **http://www.nerc-molgen.org/**
[4] NERC Data GRID: **http://ndg.nerc.ac.uk/**
[5] National Institute for Environmental eScience: **http://www.niees.ac.uk/**
[7] Environment Canada: **http://www.ec.gc.ca/envhome.html**
[8] AQUAFIRST: **http://aquafirst.vitamib.com/**
[9] MIAME/Env: **http://envgen.nox.ac.uk/miame/miame_env.html**
[10] EcoGRID: **http://www.e-science.clrc.ac.uk/web/projects/ecological_data_grid**
[11] EnvBase: **http://envgen.nox.ac.uk/public_catalogue.php**

### References

Bolhuis H, Poele EM, Rodriguez-Valera F. 2004. Isolation and cultivation of Walsby's square archaeon. *Environ Microbiol* **6**: 1287–1291.

Field D, Garrity G, Morrison N *et al.* 2005. eGenomics: Cataloguing our Complete Genome Collection.

Field D, Hughes J. 2005. Cataloguing our current genome collection. *Microbiology* **151**: 1016–1019.

Field D, Tiwari B, Snape J. 2005. Bioinformatics and data management support for environmental genomics. *PLoS Biol* **3**: e297.

Hancock D, Wilson M, Velarde G, *et al.* 2005. maxdLoad2 and maxdBrowse: standards compliant tools for microarray experimental annotation, data management and dissemination. *BMC Bioinformatics* (submitted).

Johnston AW, Li Y, Ogilvie L. 2005. Metagenomic marine nitrogen fixation — feast or famine? *Trends Microbiol* **13**: 416–420.

Pinney JW, Shirley MW, McConkey GA, Westhead DR. 2005. metaSHARK: software for automated metabolic network prediction from DNA sequence and its application to the genomes of *Plasmodium falciparum* and *Eimeria tenella*. *Nucleic Acids Res* **33**: 1399–1409.

Tiwari B, Field D. 2005. The Bioinformatics Playground. *LinuxUser Developer* **46**: 50–56.

Copyright © 2006 John Wiley & Sons, Ltd.
